# Successful Autotransplantation of a Mandibular Third Molar to Replace a Non-restorable Mandibular First Molar: A Case Report

**DOI:** 10.7759/cureus.93067

**Published:** 2025-09-23

**Authors:** Rahul Deore, Kranthikumar Reddy, Prashant Rajput, Saurabh Patil, Rutuja Malashetti

**Affiliations:** 1 Department of Conservative Dentistry and Endodontics, Jawahar Medical Foundation's Annasaheb Chudaman Patil Memorial Dental College, Dhule, IND

**Keywords:** autologous, mandibular third molar, printed, replica, three-dimensional, transplantation

## Abstract

This case report describes the successful autotransplantation of a mandibular left third molar (tooth 38) to replace a non-restorable mandibular left first molar (tooth 36) in a 38-year-old female patient who presented with severe pain due to extensive caries and periapical pathology. Preoperative intraoral periapical radiographs via radiovisiography (RVG) confirmed the suitability of tooth 38 as a donor owing to its vitality and compatible root morphology. The procedure involved atraumatic extraction of tooth 36, precise socket preparation using a 3D-printed replica, and immediate transplantation of tooth 38 under local anesthesia. A non-rigid splint stabilized the autotransplanted tooth, and elective root canal treatment was performed at two weeks to prevent pulp necrosis. Follow-up at one, three, six, and 12 months revealed clinical stability, normal occlusion, and radiographic evidence of periodontal ligament healing, without resorption or ankylosis. The patient reported restored function and satisfaction. This case highlights autotransplantation as a viable and cost-effective alternative to implants, preserving natural dentition in adults with mature donor teeth, and underscores the importance of digital planning for optimal outcomes. A long-term follow-up is recommended to ensure sustained success.

## Introduction

Tooth autotransplantation, the surgical relocation of a tooth from one intraoral site to another within the same individual, represents a biologically conservative approach to tooth replacement [1]. First described in the mid-20th century, this technique has evolved significantly with advancements in imaging, surgical precision, and periodontal management, achieving success rates of 90-95% for molars when performed under optimal conditions [2,3]. It is particularly indicated in cases of non-restorable teeth due to extensive decay, trauma, or congenital absence, where a suitable donor tooth, often an impacted or supernumerary third molar, is available [1]. Unlike prosthetic replacements such as implants or bridges, autotransplantation preserves pulpal vitality, maintains alveolar bone integrity, and promotes natural occlusal function, thereby minimizing long-term complications, such as peri-implantitis or abutment failure [4]. Third molars, with their underdeveloped root apices in younger patients, are ideal donors because of their enhanced healing potential; however, successful outcomes have been reported even in adults up to 66 years of age, challenging earlier age-related contraindications [3,5].

The procedure typically involves atraumatic extraction of the recipient tooth, precise preparation of the socket to accommodate the donor tooth morphology, and immediate replantation to ensure periodontal ligament (PDL) viability [1]. Factors influencing success include minimal extraoral time (ideally under 30 minutes), donor tooth selection based on root development (stages 3-4 on Moorrees' scale), and postoperative splinting for seven to 14 days to stabilize the transplant while allowing revascularization [6]. Complications such as root resorption, ankylosis, or pulp necrosis can occur, but are mitigated through endodontic therapy if needed, often electively performed two to four weeks post-transplantation [7]. In the mandibular arch, autotransplantation of third molars to replace the first or second molars has shown promising results, with radiographic evidence of continued root development and functional integration over the follow-up period [8].

This case report aimed to demonstrate the efficacy of immediate mandibular third molar autotransplantation as a viable, patient-centered alternative for replacing a non-restorable mandibular first molar in an adult patient. This highlights the procedure's role in preserving natural dentition, promoting alveolar bone integrity, and improving quality of life, while emphasizing key procedural considerations to optimize clinical outcomes in a 38-year-old female patient.

## Case presentation

A 38-year-old female presented to the Department of Conservative Dentistry and Endodontics, Jawahar Medical Foundation’s Annasaheb Chudaman Patil Memorial Dental College, Dhule, India, in January 2024 with a chief complaint of pain and food impaction in the lower left posterior region for three weeks. The patient reported intermittent dull pain exacerbated by chewing, with no significant medical history or systemic conditions. She was a non-smoker with good oral hygiene.

**Figure 1:**
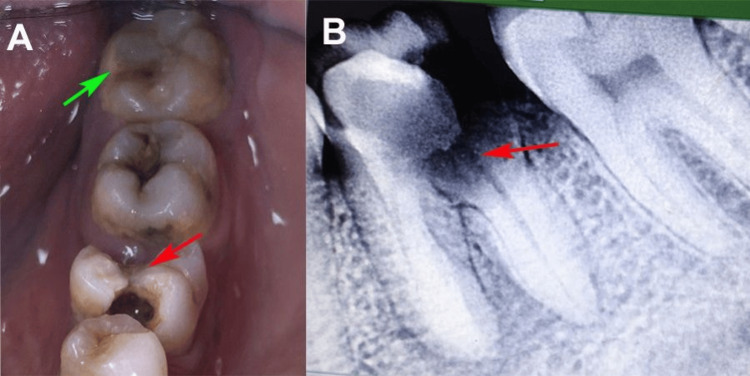
(A) Intraoral image showing grossly decayed left mandibular first molar (red arrow) and intact healthy third molar (green arrow). (B) Intraoral periapical radiograph showing grossly decayed left mandibular first molar (red arrow).

Intraoral examination revealed a grossly decayed mandibular left first molar (tooth 36) with extensive caries involving the distal and occlusal surfaces extending into the pulp chamber. The tooth was tender on percussion, with a probing depth of 6 mm on the distal aspect, and signs of gingival inflammation. The mandibular left third molar (tooth 38) was fully erupted, caries-free, and vital with a probing depth of 3 mm and no mobility. Occlusal analysis confirmed adequate space for autotransplantation, with mesiodistal dimensions of tooth 38 compatible with the recipient site (Figure 1A).

A preoperative panoramic radiograph, intraoral periapical radiograph (radiovisiography (RVG), Carestream Dental, Atlanta, GA), and cone-beam computed tomography (CBCT) scan (CS 9300, Carestream Dental, Atlanta, GA) were obtained. An extensive radiolucency involving the pulp and periapical region of tooth 36, suggestive of irreversible pulpitis and chronic apical periodontitis, was revealed (Figure 1B). Tooth 38 exhibited complete root formation, with two roots, no caries, and no periapical pathology. The CBCT provided a detailed assessment of bone width and height at the recipient site (tooth 36), confirming adequate alveolar bone volume and no proximity to the mandibular canal, ensuring feasibility for transplantation. Tooth 36 was diagnosed as non-restorable owing to extensive caries, irreversible pulpitis, and symptomatic apical periodontitis. Tooth 38 was deemed a suitable donor because of its vitality, compatible morphology, and lack of pathology.

**Figure 2:**
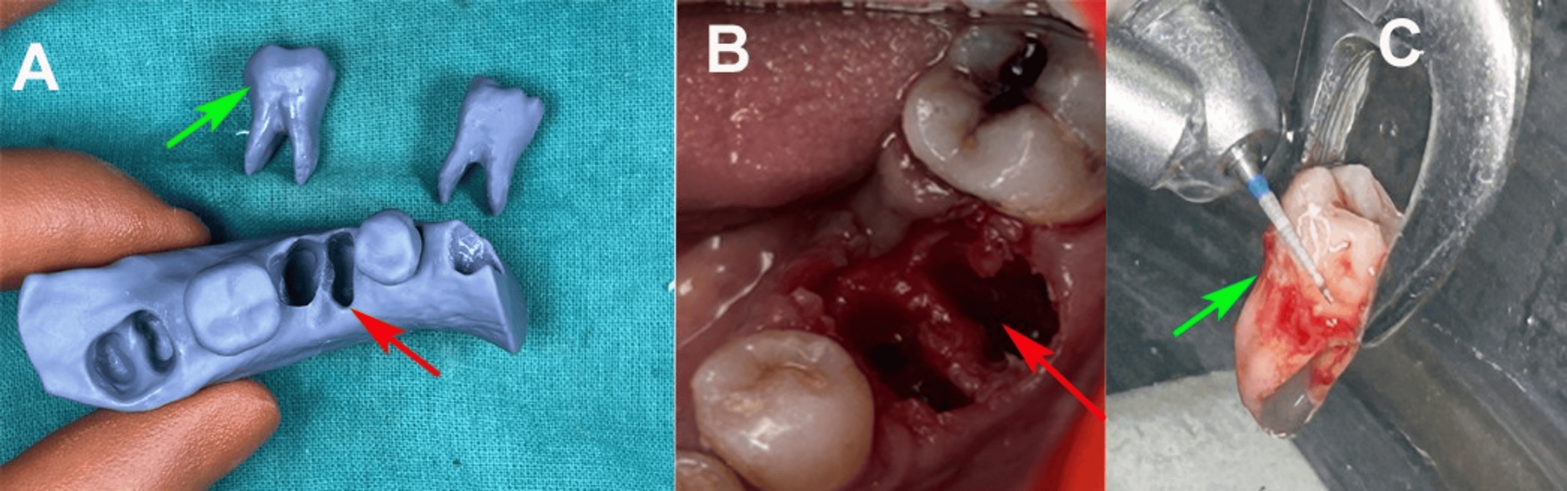
(A) 3D-printed replica of extraction socket (red arrow) and left mandibular first (green arrow) and third molar. (B) Extraction socket of left mandibular first molar for immediate autotransplantation (red arrow). (C) Preparation of extracted left mandibular third molar for autotransplantation (green arrow).

The treatment plan involved the extraction of tooth 36, immediate autotransplantation of tooth 38, and postoperative stabilization. The CBCT data were imported into digital planning software (Mimics Innovation Suite, Materialise, Leuven, Belgium) to segment the donor tooth (tooth 38) for precise 3D modeling. A three-dimensional (3D) printed replica of tooth 38 was fabricated using a 3D printer (Form 3, Formlabs, Somerville, MA) based on the segmented CBCT data (Figure 2A). A surgical guide (V Print Splint Clear, VOCO GmbH, Cuxhaven, Germany) was also fabricated using digital planning software (BlueSkyPlan, Blue Sky Bio, Libertyville, IL) to ensure precise socket preparation and minimize extraoral time. One week prior to surgery, the patient underwent a comprehensive dental hygiene assessment, including scaling and root planing, to optimize oral conditions.

The procedure was performed under local anesthesia (2% lidocaine with 1:80,000 epinephrine; Huons Co., Seongnam, South Korea). Tooth 36 was atraumatically extracted using dental elevators and forceps to preserve its alveolar socket (Figure 2B). The socket was curetted and irrigated with 0.9% normal saline (Alkem Laboratories, Ltd., Mumbai, India). The 3D replica of the donor teeth was sterilized with ethylene oxide before surgery. A 3D-printed replica was used to adjust the recipient socket with a slow-speed surgical round bur (SS White, Lakewood, NJ) under saline irrigation to match the root morphology of the donor tooth.

**Figure 3:**
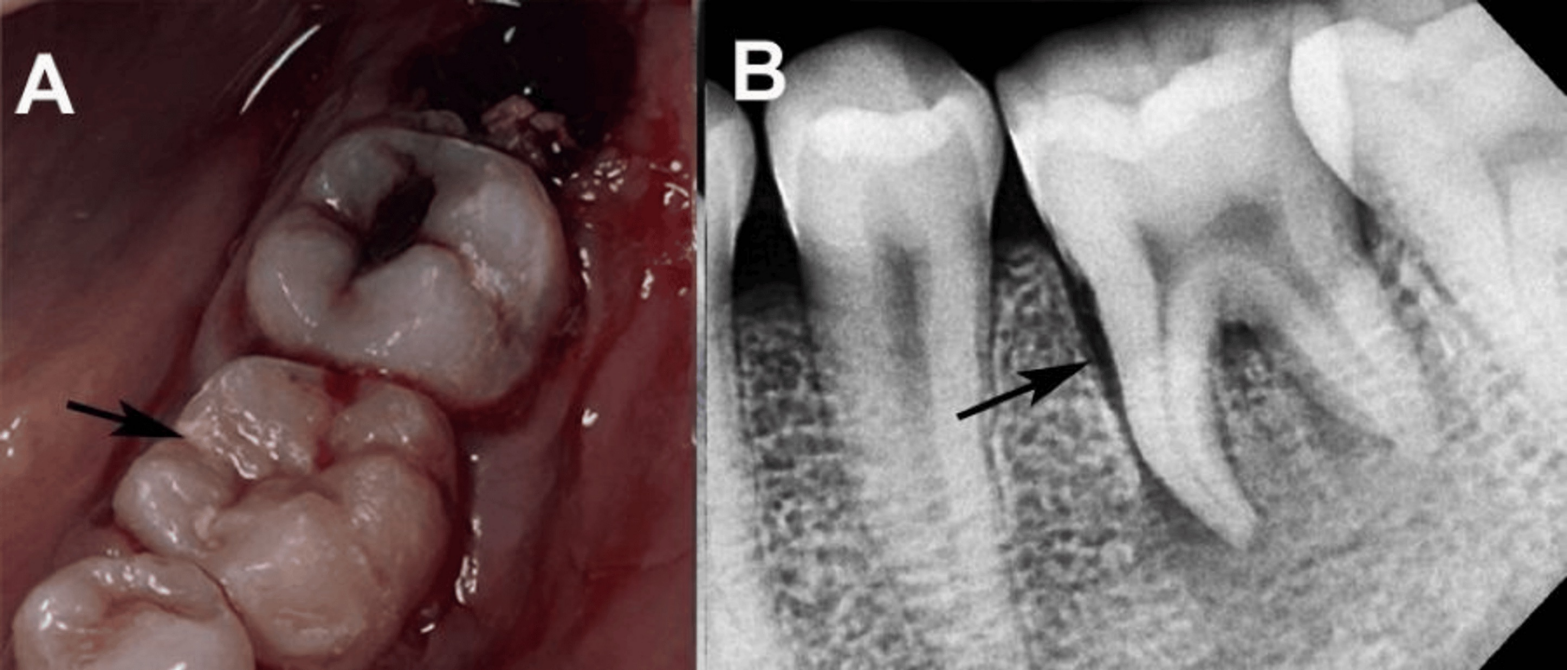
(A) Left mandibular third molar autotransplanted in the socket of left mandibular first molar (black arrow). (B) Intraoral periapical radiograph analyzing correct position of the transplanted tooth in socket (black arrow).

Tooth 38 was carefully extracted, preserving the PDL, and stored in sterile saline for less than five minutes (Figure 2C). The donor tooth was positioned in the prepared socket of tooth 36 under light pressure to ensure fit, guided by a 3D-printed surgical template. Occlusal interferences were adjusted extraorally using a high-speed handpiece (NSK, Tokyo, Japan). The transplanted tooth was stabilized with a non-rigid splint using a malleable orthodontic wire (Tru-Arch, Ormco Corporation, Orange, CA) and composite resin (Filtek Z250, 3M, St. Paul, MN) extending from tooth 35 to the transplanted tooth. The flap was sutured with 3-0 silk sutures (Sutures India Pvt. Ltd., Bengaluru, India) for close approximation (Figure 3).

Postoperative instructions included a soft diet, 0.12% chlorhexidine gluconate rinse (Colgate-Palmolive, NY) twice daily for seven days, and antibiotic prophylaxis (amoxicillin 500 mg three times daily, Alkem Laboratories Ltd.) for five days. Endodontic treatment was planned to mitigate the risk of pulp necrosis.

Two weeks after transplantation, root canal treatment was performed on the transplanted tooth 38. Access was obtained under rubber dam isolation (Sanctuary Dental Dam, Sanctuary Health, Malaysia), and canals were prepared using ProTaper Next files (Dentsply Sirona, Charlotte, NC). Irrigation was performed using 2.5% sodium hypochlorite (Parcan, Septodont, Saint-Maur-des-Fossés, France) and 17% ethylenediaminetetraacetic acid (CanalPro, Coltene, Altstätten, Switzerland). Canals were obturated with gutta-percha (Dentsply Sirona) and AH Plus sealer (Dentsply Sirona). The final restoration was performed using a Filtek Z250 composite (3M).

**Figure 4:**
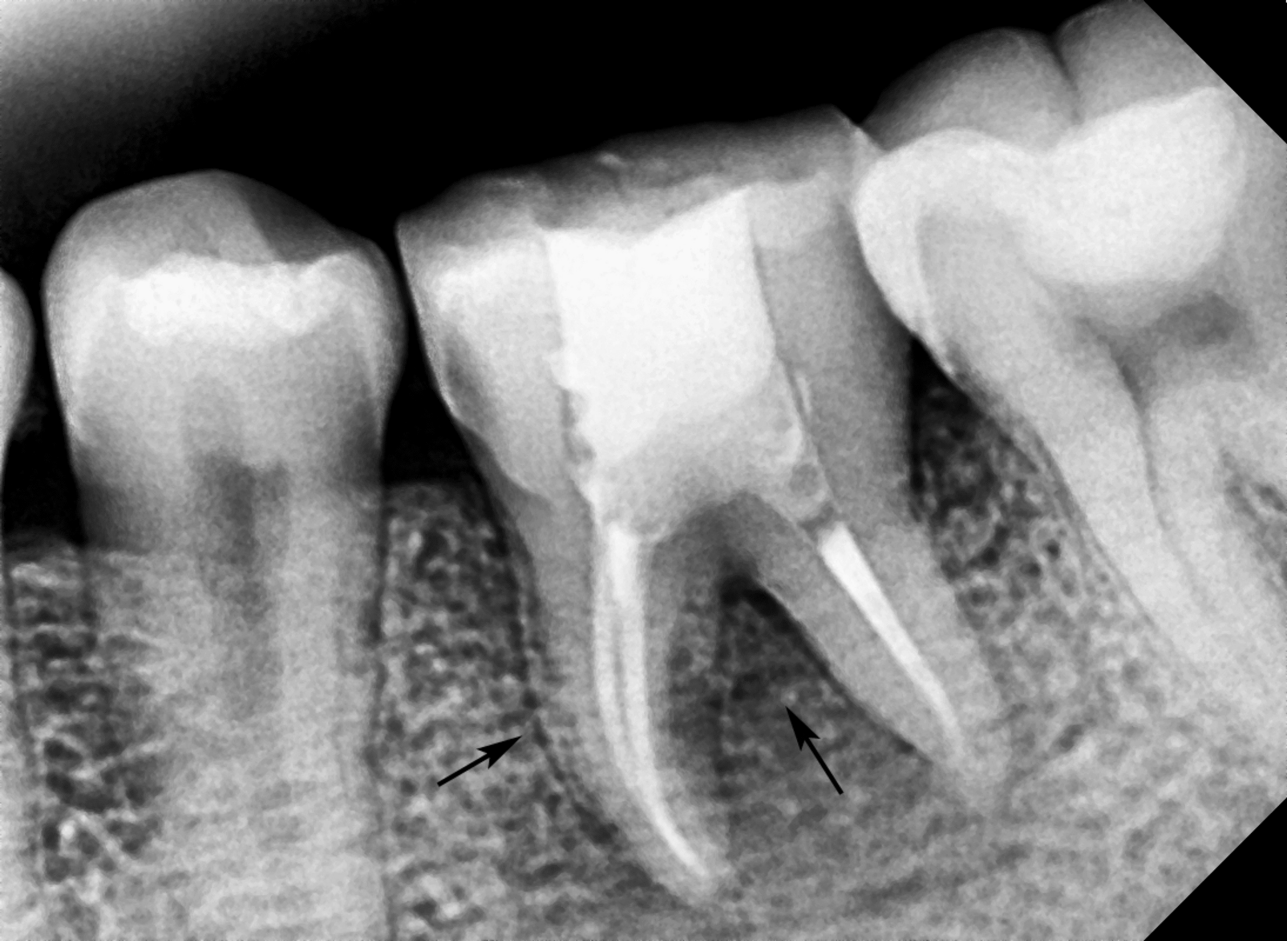
Postoperative intraoral periapical radiograph after six months showing root canal-treated autotransplanted tooth, healed periapical area with periodontal ligament space (black arrows).

The patient was reviewed at one week, one month, three months, six months, and 12 months after transplantation. At one week, suture removal revealed satisfactory soft-tissue healing. At one month, the splint was removed, and the transplanted tooth exhibited grade 1 mobility within normal limits. Clinical examination showed no tenderness, with a probing depth of 3 mm and healthy gingival margins. Radiographic evaluation at three months demonstrated reorganization of the periodontal space with no signs of root resorption or ankylosis. At six months, the tooth was fully functional with normal occlusion and no masticatory dysfunction (Figure 4). The 12-month follow-up confirmed stable periodontal health, absence of mobility, radiographic evidence of bone regeneration, and normal PDL space. The patient reported satisfaction with the aesthetics and function.

## Discussion

This case report presents the successful autotransplantation of a mature left mandibular third molar to replace a non-restorable first molar in a 38-year-old female patient, achieving clinical and radiographic stability at 12 months without complications, such as root resorption or ankylosis. Tooth autotransplantation, particularly involving third molars, has been increasingly recognized as a biologically sound alternative to implants or fixed prosthodontics, preserving natural tooth structure and periodontal health [1,8]. Historical data indicate that early attempts in the 1950s yielded success rates of approximately 50%; however, advancements in atraumatic extraction techniques, digital planning, and postoperative management have increased overall survival rates to 90-98% at one to five years, with success rates (defined as functional integration without pathology) reaching 81-95% for mature teeth [9].

In this case, a short extraoral time of less than five minutes and preservation of the PDL align with key prognostic factors identified in the literature [1,8], where minimizing extraoral exposure to < 10 minutes correlates with reduced inflammatory root resorption and higher pulp survival. This was made possible by using a 3D-printed replica of the donor tooth. This is supported by a previous study [10] and systematic review [11], where the use of 3D-printed replicas significantly reduced the extraoral time, thereby increasing the success rate.

A comparative analysis with prior case reports underscores the feasibility of third molar autotransplantation in adults, even with fully formed roots, challenging earlier contraindications limited to adolescents [2,5,8]. For instance, a 2024 case report detailed the successful transplantation of a third molar to replace a second molar in a 66-year-old patient, reporting 100% periodontal healing at 18 months, similar to our findings of normal probing depths and absence of mobility at one-month post-procedure [2]. Another case report on immediate autotransplantation of mature third molars to bilateral first molar sites with periapical lesions achieved a 92% success rate over a mean 47-month follow-up [5], emphasizing the role of preoperative radiographic assessment. As in our case, CBCT imaging confirmed compatible morphology and bone support, facilitating precise socket adaptation.

Success rates for immature third molars are notably higher at 94-97% over 10 years, but mature tooth outcomes, as in our patient, hover around 89-91%, influenced by elective endodontic intervention [7]. In this report, root canal therapy performed two weeks post-transplantation prevented potential pulp necrosis, a common complication in 20-30% of mature transplants [1]. Dhar et al. [8] conducted a study on 20 adult patients in which fully mature mandibular third molars were autotransplanted in mandibular first molar extraction sites, and all patients were monitored for one year. It was noticed that only two out of 20 cases failed due to root resorption in one case and insufficient bone in the mandibular first molar region in the second case. In our case, the bone quality in the region of tooth 36 was checked before autotransplantation and found to be satisfactory.

Long-term studies further validated our 12-month radiographic evidence of PDL reorganization and bone regeneration [8,10]. A prospective evaluation of mandibular third molar autotransplants in freshly extracted mandibular first molars reported 95.2% survival at 10 years, attributing durability to non-rigid splinting and antibiotic prophylaxis, elements employed here to mitigate infection risk at the inflamed recipient site [10]. Wu et al. [10] reported a 100% success rate of autotransplanted third molars using a 3D replica of donor teeth at a year’s follow-up. Lee et al. [12] reported that the average deviations of a replica model produced by a 3D printer ranged from 0.038 to 0.047 mm, which is considerably lower than the clinically permissible threshold. Our patient's rapid functional recovery, with normal occlusion by six months, aligns with these findings, where positive pulp responses post-endodontics reached 90-95% by eight weeks in comparable cohorts [8]. However, variations in success, such as 61-100% across reports, stem from differences in donor vitality and surgical expertise, highlighting the need for case selection based on vitality testing and atraumatic techniques [6]. The efficacy of this procedure in adults has expanded its applicability beyond pediatric cases. Nonetheless, while our outcomes are promising, they reflect a single case with a relatively short follow-up period compared to the multi-year series.

Clinical implications include promoting third molar autotransplantation as a cost-effective, tissue-preserving option for non-restorable molars in adults with suitable donors, potentially reducing reliance on implants, and enhancing dentition longevity. Limitations include the absence of long-term data, necessitating larger prospective trials for broader generalizability.

## Conclusions

Autotransplantation of the mandibular third molar to replace a non-restorable first molar in a 38-year-old female was successful with clinical and radiographic stability at 12 months. Using CBCT imaging and 3D-printed surgical guides, the procedure achieved a precise socket fit and minimal extraoral time. Endodontic treatment at two weeks prevented pulp necrosis and non-rigid splinting-aided healing. Follow-up showed normal occlusion and function, and no signs of resorption or ankylosis. This case demonstrated that autotransplantation is an effective biological alternative to implants for adults with suitable donor teeth. Continuous monitoring is advised to ensure long-term success and patient satisfaction.
